# A fluidics-based impact sensor

**DOI:** 10.1371/journal.pone.0195741

**Published:** 2018-04-10

**Authors:** Daigo Takahashi, Keisuke Hara, Taiji Okano, Hiroaki Suzuki

**Affiliations:** Faculty of Science and Engineering, Chuo University, Tokyo, Japan; Institute of Materials Science, GERMANY

## Abstract

Microelectromechanical systems (MEMS)-based high-performance accelerometers are ubiquitously used in various electronic devices. However, there is an existing need to detect physical impacts using low-cost devices with no electronic circuits or a battery. We designed and fabricated an impact sensor prototype using a commercial stereolithography apparatus that only consists of a plastic housing and working fluids. The sensor device responds to the instantaneous acceleration (impact) by deformation and pinch off of a water droplet that is suspended in oil in a sensor cavity. We tested the various geometrical and physical parameters of the impact sensor to identify their relations to threshold acceleration values. We show that the state diagram that is plotted against the dimensionless Archimedes and Bond numbers adequately describes the response of the proposed sensor.

## Introduction

Silicon-based solid-state accelerometers are ubiquitously used nowadays in various consumer electronic devices such as automobiles, smartphones, video games, and wearable equipment [1–5]. Because MEMS devices are well suited for integration in electronic circuits, physical acceleration data converted to electronic signals are often processed by the computer, stored in memory, and transmitted to other devices, depending on the applications [6–8].

However, there is a need for a device to detect acceleration in an easier manner and at a lower cost without the use of electronics or a battery. For instance, in logistics, customers want to know whether their packages have been delivered safely and that they have not been dropped or crushed. Modern MEMS accelerometers are equipped with a data logger and memory, but they are still costly, and they have been only used for the delivery of special items. Another need for an accelerometer is in sports. In many contact sports, injuries (e.g., head injuries, concussions) often arise from physical contacts, such as in American football [9], rugby [10, 11], ice hockey [12, 13], basketball, soccer [14, 15], judo [16], and many others. Today, MEMS-based sensors have been developed specifically for these sports [17], and are sold in the market [11, 18, 19]. These are used to monitor impacts mostly in well-funded professional and top-level college sports [12, 13, 20, 21]. When a severe impact occurs on an athlete’s head, the player is withdrawn from the game or practice, and is advised to undergo a medical check. Correspondingly, these sensors can be also utilized for gathering statistical data for impacts during sports, which are then analyzed to allow enforcement of appropriate safety measures [22–25]. Considering that in many sports the practice and game plans are decided based on experimental grounds, statistical data should be gathered and used even in recreational, amateur, high-school, youth, and children's sports [15, 19, 26–30]. MEMS wearable devices specialized for sports are still too expensive to be used by the majority of players, or in every sports scene [17, 18, 20, 31].

For these destined applications, precision in the recorded acceleration values, or the data time resolution, need not be very high. It is often sufficient to know whether or not a recorded acceleration surpasses a certain threshold value. In transportations, use of such a simple and low-cost device ensures the safety of the package throughout its logistical processing. In sports, it becomes possible to comprehend the statistics of dangers associated with daily practices and games and to encourage a player who has experienced jolts to seek medical care.

In this work, we propose a simple binary sensor that only consists of a plastic housing and working fluids to detect instantaneous acceleration (impact). In this sensor, a water droplet serves as the inertial proof mass. The droplet is suspended in oil and is deformed by acceleration, thereby allowing it to be eventually pinched off. Several research studies have been conducted to develop fluidics-based inertial sensors, but they still use complicated structures and electronic readouts [32–35]. Herein, we fabricated an extremely simple impact sensor in which the readout is based on the visual judgment, using a commercial stereolithography apparatus. No electronic circuit or battery is necessary so that it can be cost-effective and process-effective in mass production, leading to its widespread use in various applications. We show that our prototype sensor was able to detect the g values ranging from 30 to 150 g with a duration of ~10 ms. By changing the physical parameters of its design and working fluids, we investigated the physical principles underlying the function of the fluidics-based sensing device.

## Sensing principle

**Fig 1:**
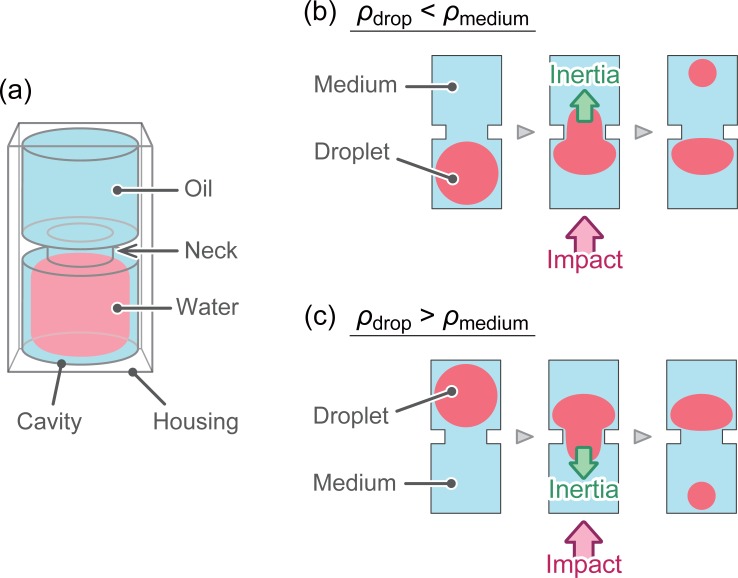
Schematics of the fluidic based impact sensor. (a) Schematic overview of the basic structure of the sensor. (b, c) Working principle of the sensor based on which impacts are detected depending on different combinations of immiscible working fluids.

We fabricated the rectangular housing with a hollow cylindrical cavity with a narrow neck (constriction) in the middle, as seen in Fig 1(A). This cavity is filled with oil, and a water droplet immersed in it serves as a proof mass. The droplet is carefully placed on one side of the neck. Owing to the interfacial tension, the water droplet does not readily move across the neck when the device is at rest. When an acceleration or an impact is exerted on the device parallel to the axis of the cylindrical cavity, an inertial force is exerted on the water droplet relative to the surrounding oil as there is a density difference between oil and water. As a result, part of the water droplet deforms and enters into the constriction. When the acceleration is large enough, the water droplet is elongated and is pinched off. Herein, the direction of the droplet movement depends on the density difference. When a hydrocarbon-based oil is used (relative density *ρ*_medium_ = 0.6−0.8 g/L), the droplet moves toward the direction of the impact (Fig 1B). However, when fluorocarbon-based oil (*ρ*_medium_~1.8 g/L) is used, the droplet moves in a direction opposite to the acceleration (Fig 1C). The separated small droplet moves to the other side of the constriction and is readily detected by human eyes. The threshold for the movement of the droplet depends on various physical parameters, such as the diameter of the constriction, density difference, interfacial tension between fluids, and viscosity of fluids.

## Materials and methods

### Fabrication process

**Fig 2:**
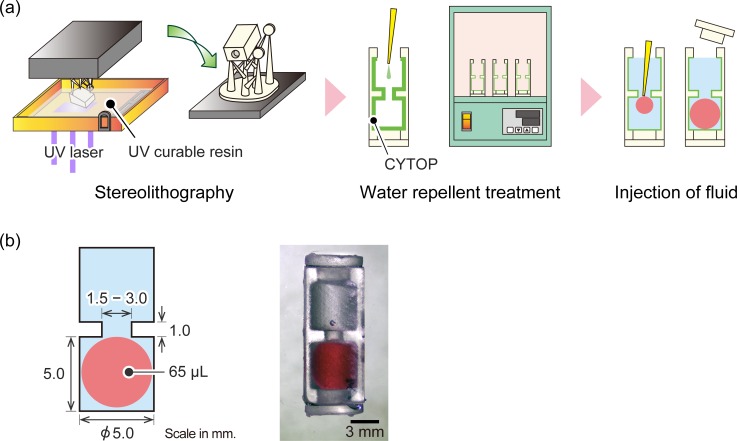
Fabrication and design of the impact sensor. (a) Fabrication process flow. (b) Actual design in cross section, and an image rendition of the complete sensor.

We fabricated the housing of the prototype device using a commercial stereolithography apparatus (Form2, Formlabs) equipped with a 405-nm violet laser (*φ* = 140 μm laser spot) and a layering thickness of 50 μm, according to the manufacturer’s protocol. The process flow is depicted in Fig 2(A). In short, we designed the device with a 3D CAD software (Rhinoceros 5.0). The design was saved in a file which was converted in the .stl format for processing by the apparatus. The device consisted of a main housing with a hollow cylindrical cavity and two lids. After the lithography process, the device was removed from the stage and uncured resin was washed with isopropanol. After drying, supporting pillars were removed manually using nippers. After closing one side of the cavity using one of the lids, we applied the amorphous fluorinated polymer CYTOP in the internal wall of the cavity to make it highly hydrophobic. It was completely dried by baking at a temperature of 160° for 1 h in the furnace. We confirmed that the contact angle (CA) of the native resin in air increased from 71° to 96° after the CYTOP treatment.

We then injected the fluorocarbon oil and 65 μl of colored water using a pipette. We then sealed another side of the cylinder using another lid carefully so that the air bubble was not trapped in the cavity. In the initial stage of the development, we also tested the hydrocarbon oil (such as mineral oil and liquid paraffin), but found that the fluorocarbon was more suitable because it had a large density difference from water. Thus, we employed commercial fluorocarbon oil (FC-40 and FC-770, 3M) as the basic surrounding medium.

The outer dimensions of the device are 6 × 6 × 16 mm^3^ and have two hollow cylindrical cavities with a 5-mm inner diameter and a 5-mm height on both sides (Fig 2B). These large cavities are connected by a narrow channel (neck or constriction) with an inner diameter of 1.5−3.0 mm and a length of 1 mm. A photograph of the completed device is shown in Fig 2(B).

### Falling test apparatus

**Fig 3:**
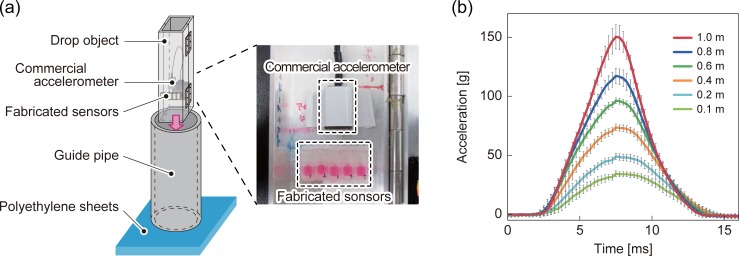
Falling test apparatus. (a) Custom-made apparatus consisting of drop object, guide pipe, and polyethylene sheet. (b) Temporal acceleration profiles of the object dropped from different heights (0.1−1.0 m) measured by the commercial accelerometer.

We tested the response of our fluid-based impact sensor in accordance to the falling test using the custom-made setup shown in Fig 3(A). We fixed our sensors together with the commercially available three-axis MEMS accelerometer—equipped with Wi-Fi data transmission (MA3, Microstone, Japan)—to the metal case that consisted of two pieces of channel-type aluminum bars connected by a hinge. This object (dimensions: 50 × 50 × 370 mm, total mass: 600 g) was subjected to a free fall through a 75-mm inner diameter PVC pipe (guide pipe). Because the diagonal size of the housing was 70.7 mm, the housing fell vertically. The object fell from various heights onto a 20-mm thick polyethylene sheet.

MEMS accelerometers that are designed for contact sports that are available in the market are designed to detect accelerations above 50 g, and most of the published research papers on this topic indicate that accelerations higher than approximately 100 g can possibly cause traumatic head injuries or concussion [20, 21, 36, 37]. Thus, we decided to set the detection range of our sensor to 30−150 g.

Prior to the experiment, we evaluated our handmade apparatus. The raw signal generated from the commercial accelerometer, averaged over five independent falling experiments at each height, is shown in Fig 3(B). This result shows that our falling test apparatus is able to generate reproducible acceleration profiles in the acceleration range of 30−150 g within a time period of ~10 ms. This period is defined as the time difference between the rising and falling signal edges at 0 g (the peak acceleration value and the duration of each falling height are shown in S1 Fig). This duration is also a typical value reported in contact sports, such as in American football hits [20, 30, 38], soccer head contacts [39], and judo waza [16] in accordance to the literature.

## Results and discussion

There are several parameters that determine the threshold of impact detection. As wider the diameter of the neck and the strength of the interfacial tension of immiscible fluids decrease, the deformation of the water droplet also increases. As the density difference between immiscible fluids increases, the relative inertia force increases. Additionally, as the viscosity values of the fluids are increased, the deformation of the water droplet occurs within a shorter time period. We carefully studied the influence of each of these parameters, as outlined below.

### Effect of neck diameter

**Fig 4:**
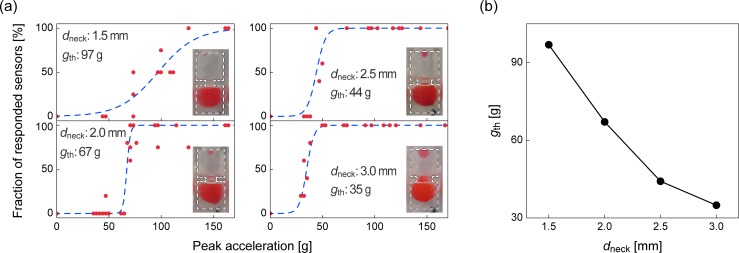
Result of the impact sensors with different neck diameter *d*_neck_. (a) Fraction of responded sensors versus peak acceleration values with *d*_neck_ = 1.5−3.0 mm. Insets show typical images of responded states after droplet falls. Blue dashed lines represent the fitted curves based on the use of the sigmoidal function. (b) Plot showing the dependence of *g*_th_ on *d*_neck_.

We tested the inner diameters of the neck *d*_neck_ to be 1.5, 2.0, 2.5, and 3.0 mm. When the droplet remained unchanged after a fall, we considered that the sensor did not respond. When at least one part of the droplet separated from the “mother droplet” and moved to the other side of the neck, we considered that the sensor responded. Fig 4(A) shows the fraction of the sensors that responded when tested at various falling heights. That is, when at least one part of the water droplet moved to the other side in three of the five tested sensors, the success fraction was 60%. We repeated the falling tests at various heights, and plotted the fraction versus peak acceleration value (*g*_peak_) obtained from the MEMS accelerometer profile. The result was fitted with the sigmoidal function, which is often used for modeling the stepwise behavior; i.e.,

$$f(g_{\mathrm{peak}})=\frac{1}{1+\exp\left\{-k(g_{\mathrm{peak}}-g_{\mathrm{th}})\right\}},$$ (1)

where *k* and *g*_th_ are fitting parameters. The threshold *g*_peak_ value of the sensor was determined as the inflection point *g*_th_ of the curve. This nonlinear fitting was performed based on the least-squares-method using the solver in Microsoft Excel (see S1 Table for all parameter-fitting results). As seen in Fig 4(A), the neck diameter had a significant effect on the *g*_th_ values. The variable *g*_th_ is plotted as a function of *d*_neck_ in Fig 4(B). As the inner diameter increases, the threshold decreases. At the smallest neck diameter (*d*_neck_ = 1.5 mm) the slope of the fitting function is small, while a steep change is elicited with *d*_neck_ ≥ 2.0 mm. This result indicates that the detection becomes sensitive to small variations, and stochastic at small values of *d*_neck_. Moreover, as observed in the typical images in the insets, at *d*_neck_ = 1.5 mm, the detached droplet that moved to the other side was too small to be recognized with naked eyes. The diameter of the separated droplet was mainly depended on *d*_neck_ (S2 Fig).

The present result indicates that *g*_th_ can be easily adjusted by the neck diameter *d*_neck_, but reproducibility and visual recognition can be compromised in high impact detections at small *d*_neck_ values. Thus, we decided to employ the device using *d*_neck_ = 2.0 or 3.0 mm in the conducted experiments, as described next.

### Effect of viscosities of working fluids

When the impact was applied to the water droplet, it deformed and penetrated the neck region. The viscosities of both fluids (water and oil) are expected to affect *g*_th_ because increased viscosity restricts the deformation within a given time. The physical constants of the working fluid tested in this work are listed in Table 1.

**Table 1:** Density, dynamic viscosity, and kinematic viscosity, of working fluids [40].

	Type of fluid	Density ×10^3^ kg/m^3^	Dynamic viscosity ×10^−3^ Pa·s	Kinematic viscosity × 10^−6^ m^2^/s
**Oil phase**	FC-770	1.85	1.40	0.76
	FC-40	1.78	4.10	2.30
**Aqueous** **phase** **(droplet)**	Water	1.00	1.00	1.00
	45% sucrose	1.19	9.40	7.89
	65% sucrose	1.30	147	113
	70% sucrose	1.33	482	362
	60% glycerol	1.14	11.0	9.64
	90% glycerol	1.22	220	180
	100% glycerol	1.25	1410	1130

**Fig 5:**
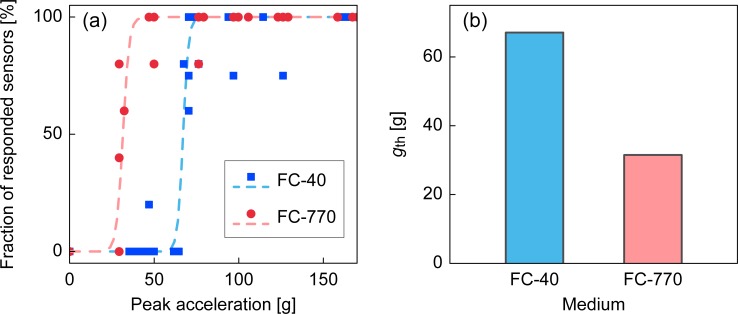
Result of the impact sensor with different oil phase. (a) Fraction of responded sensors versus peak acceleration tested using different oil viscosities. (b) Comparison of *g*_th_ for fluorocarbon oils with different viscosities.

First, we tested different fluorocarbon oils using pure water. FC-40 and FC-770 have similar densities but different viscosities (4.1 and 1.4 mPa·s, respectively). The result tested with the device with *d*_neck_ = 2.0 mm is shown in Fig 5. As expected, we obtained smaller *g*_th_ values with less viscous oils. The threefold difference of the oil viscosity yielded an approximate twofold difference in *g*_th_.

**Fig 6:**
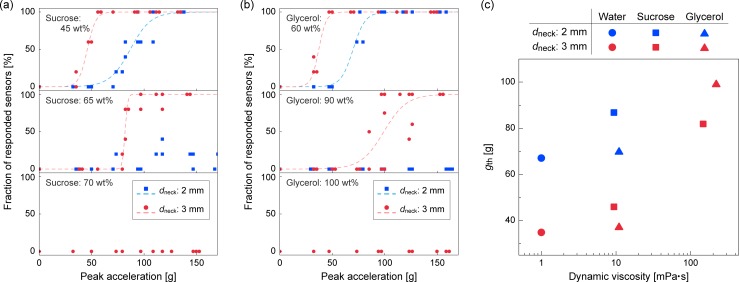
Results of the impact sensor with different aqueous solution (droplet). (a, b) Fraction of responded sensors versus peak acceleration tested at different water densities. (a) Sucrose solution (45−70 wt%), (b) glycerol solution (60−100%), and (c) dependence of *g*_th_ on water viscosity.

We subsequently tested the increased viscosity of the water droplets by adding either sugar (sucrose) or glycerol. The density and viscosity of these mixtures was obtained from the handbook, as described in Table 1. The elicited responses at *d*_neck_ = 2.0 or 3.0 mm with FC-40 as the oil phase are shown in Fig 6.

Both 45 wt% sucrose and 60 wt% glycerol aqueous solutions had viscosities that were approximately 10 times higher than pure water. Based on these tests, we obtained similar responses: *g*_th_ = 30−50 g for *d*_neck_ = 3.0 mm, and *g*_th_ = 70−90 g with *d*_neck_ = 2.0 mm (Fig 6(A) and 6(B), top). Because the *g*_th_ values of pure water are 67 g and 35 g, respectively, for 2.0 and 3.0 mm neck diameters, the effect of the water phase viscosity was not as significant as the oil viscosity. Subsequently, 65 wt% sucrose and 90 wt% glycerol solutions are more viscous than water by approximately 100 times. In these cases, the value of *g*_th_ for a device with *d*_neck_ = 3.0 mm was increased to 82 g and 99 g, respectively, whereas the fraction of the responded devices with *d*_neck_ = 2.0 mm was less than 40% when subjected to accelerations up to 150 g (Fig 6(A) and 6(B), middle). With 70 wt% sucrose solution and 100% glycerol solutions as the droplet, no sensor responded even with a *d*_neck_ value of 3.0 mm (Fig 6(A) and 6(B), bottom). These results indicate that the viscosity of the oil phase is more sensitive to the response of our sensor compared to that of the water phase. The dependencies of the *g*_th_ values on the viscosity of the water phase are summarized in Fig 6(C).

### Effect of interfacial tension

**Fig 7:**
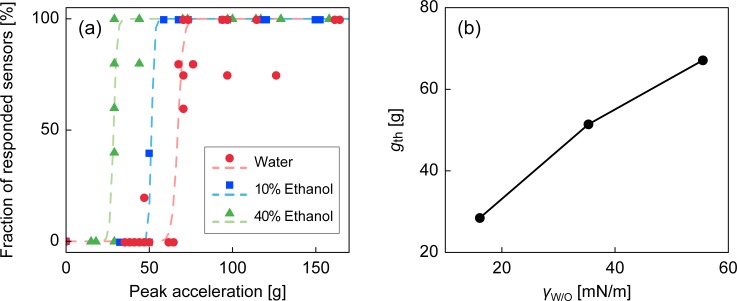
Results of the impact sensor with different interfacial tension. (a) Fraction of responded sensors versus peak acceleration tested at different interfacial tensions. (b) Dependence of *g*_th_ on *γ*_w/o_.

The extent of the deformation of the water/oil interface in response to the inertial force should also depend on the interfacial tension *γ*_w/o_. That is, larger *γ*_w/o_ values tend to maintain the water droplet spherical, and smaller *γ*_w/o_ values lead to a more elongated water droplet upon impact. We investigated this effect by varying *γ*_w/o_ between FC-40 oil and water with the addition of alcohol. Mazutis and Griffiths [41] reported that addition of 10% and 40% ethanol to the water phase reduced the interfacial tension between the fluorocarbon oil and water (originally at 55.5 mN/m) to 35.3 and 16.1 mN/m, respectively. The experimental test was conducted with a device with *d*_neck_ = 2.0 mm, and is shown in Fig 7(A). The dependence of *g*_th_ on *γ*_w/o_ (Fig 7B) shows that *g*_th_ is almost linearly dependent on *γ*_w/o_ in the tested range.

### Nondimensional analyses

Thus far, we have observed how the geometrical size of the neck and the material properties of working fluids alter the threshold acceleration value *g*_th_. In fluid mechanics, dimensionless numbers describe the overall behavior of the system. For example, the microfluidic system in which water-in-oil droplets are formed by coaxial streams are characterized by the Weber number (*Wb*, ratio of hydrodynamic force to the surface tension force) and the capillary number (*Ca*, ratio of viscous force to the interfacial tension force). [42] The state diagram of flow modes that are plotted as a function of *Wb* and *Ca*, is shown to be separated into distinct regimes describing different flow modes (jetting and dripping).

**Fig 8:**
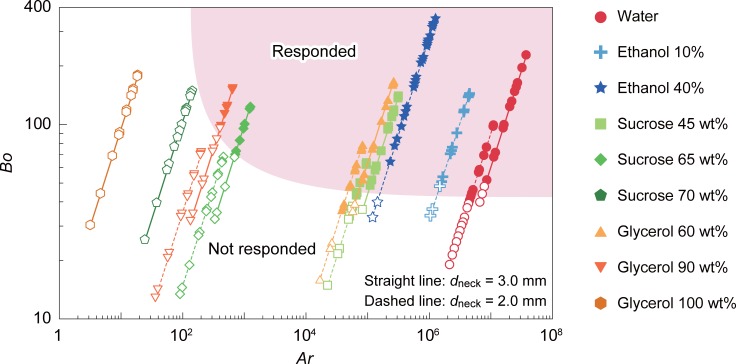
State diagram of the response of the sensor as a function of *Ar* and *Bo*. Filled symbols represent sensors that “responded,” while open symbols represent sensors that “did not respond.” Circle: water, cross: ethanol 10%, star: ethanol 40%, square: sucrose 45 wt%, diamond: sucrose 65 wt%, pentagon: sucrose 70 wt%, triangle: glycerol 60 wt%, inverse triangle: glycerol 90 wt%, and hexagon: glycerol. Straight and dashed lines represent the results obtained with *d*_neck_ = 3.0 mm and 2.0 mm, respectively.

Although seemingly similar, we found that the use of *Ca* and *Wb* in the present system was inappropriate (S3 Fig). The hydrodynamic force in *Wb* and the viscous force in *Ca* are based on the premise that flow is in a steady state. However, in the present system, the dominant force that acts during the impact is the body force that is generated instantaneously as a result of the inertia. This consideration led to the use in these analyses of the Archimedes number *Ar*, the ratio of the external body force to the viscous force, and the Bond number *Bo*, the ratio of the external body force to the interfacial tension force. The definition and usage of these variables in our system are given by the following equations,

$$Ar=\frac{\rho_{w}\Delta \rho g_{\mathrm{peak}}d_{\mathrm{neck}}^{3}}{\mu_{w}^{2}}$$ (2)

and

$$Bo=\frac{\Delta \rho g_{\mathrm{peak}}d_{\mathrm{neck}}^{2}}{\gamma_{W/O}}$$ (3)

where *ρ* and *μ* are the density and dynamic viscosity of the aqueous droplet, respectively, and *Δρ* is the density difference between water and oil. The state diagram is plotted in Fig 8, with open and filled symbols representing the conditions in which the sensors are classified as “did not respond” and “responded.” Note that we employed the viscosity of the water droplet as the viscosity *μ* in *Ar*, because it can be readily tuned by adding sugar or glycerol. However, theoretically, it could be the viscosity of oil instead. The sets of symbols connected by solid or dashed lines belong to the same device and composition, but correspond to different *g*_peak_ values (fallen heights). It is obvious that at the high *Ar* regime (10^4^ < *Ar* < 10^7^), sensors responded when *Bo* > 40. This result means that when the body force owing to acceleration is high, the response of the fluid is determined by the constant value of the ratio of the body force and the interfacial tension. In the low *Ar* regime (*Ar* < 10^2^) where the viscous drug is dominant, sensors ceased to respond because the aqueous droplet could no longer be elongated instantaneously owing to the strong damping effect. The regime where the sensors respond is highlighted in the figure.

## Conclusion

We showed that our simple impact sensor that was implemented without the use of electronics but with the use of plastic housing and working fluids only, can detect impacts (instantaneous accelerations) above certain thresholds. We fabricated and tested the prototype sensor, and established the basic design and choice of the working fluids so that the sensor covers the 30 to 100 g range that is relevant and applicable to human body or head impacts in various sports. We systematically tested the effect of the neck size and fluid parameters (density, viscosity, and interfacial tension), and discussed how these parameters influenced the threshold value. Analysis of the elicited results based on nondimensional numbers provided a useful guideline for the design of the sensor for various applications. Among these parameters, the viscosity of the aqueous droplet was the easiest controllable parameter since it could be readily tuned by additives. The viscosity of the oil could be a possible choice, but it depended on the availability of the types of oil with various viscosities. Hydrocarbon oil and silicone oils have various chain lengths and viscosities, but their densities were close to water (~0.7 g/ml and ~1.0 mg/ml, respectively). This property makes them less attractive because it reduces the relative inertial force on water. The size of the neck was also an easily changeable parameter, but it directly changed the size of the droplet after it pinched off. If its size was too small, the visual judgment was more difficult. The interfacial tension value could be the best choice since it can be tuned using surfactants. However, we did not test these variables in the present work because of the difficulty encountered in obtaining good surfactants for fluorocarbon oil. The long-term stability of the water/oil interfacial tension value could also be a problem in terms of its practical use.

Although we established that the basic design and physical mechanism of operation of our proposed sensor, there are still many issues that have to be considered. One of them is the directionality of sensing. A water droplet immersed in fluorocarbon oil tends to float so that it is always in contact with the neck cavity. However, when one aims to detect the acceleration in a direction that is not parallel to gravity, then one cavity has to be completely packed with water droplets so that the interface is always in contact with the entrance of the neck. Although we only tested the single-axis sensor, multidirectional detection could be implemented by the appropriate design of the housing. Connecting the donut-shape cavity to the center cavity with a droplet will allow this device to be able to detect 2D impacts. Another important issue is the shelf life. The packaging and hermeticity of our handmade prototype was imperfect, and an air bubble gradually grew in the cavity within several days that deteriorated the reproducibility of the detection. Mass production with the plastic molding and automated fluid injection system should solve this problem. We are currently working on these issues to develop a more practical version of this impact sensor.

Although it is still in a primitive stage, we believe that the development of this extremely low-cost impact sensor without the use of electronics would benefit certain real-life applications, such as sports and logistics. It is envisaged to be particularly useful and important in sports where quantitative data can be obtained to evaluate safety. The appropriate safety measure should be enforced for professional and high-level players and for amateurs, including players at younger ages. Our efforts to identify suitable usages and appropriate configurations in practical applications are ongoing.

## Supporting information

S1 Fig Characterization of the custom-made falling apparatus. (a) Dependence of the peak acceleration values on the drop height. (b) Dependence of the duration of acceleration profile on the drop height.
(EPS)

S2 Fig Scatter plot of the diameter of separated droplets. Diameters measured in each falling experiments with different neck diameters are plotted versus each acceleration value.
(EPS)

S3 Fig State diagram of the response of the sensor as a function of Reynolds number and Weber number. This plot shows inconsistent responses among different experimental conditions but within similar regions.
(EPS)

S1 Table Parameters of curve fitting results for all experimental data presented in Figs 4–7. (XLSX)
